# *N*-Methyl­pyrrolidine-1-carbothio­amide

**DOI:** 10.1107/S1600536812020971

**Published:** 2012-05-12

**Authors:** M. Naveed Umar, M. Nawaz Tahir, Mohammad Shoaib, Akbar Ali, Imran Khan

**Affiliations:** a Department of Chemistry, University of Malakand, Pakistan; b University of Sargodha, Department of Physics, Sargodha, Pakistan; c Department of Pharmacy, University of Malakand, Pakistan; d Department of Biotechnology, University of Malakand, Pakistan

## Abstract

There are two independent mol­ecules in the asymmetric unit of the title compound, C_6_H_12_N_2_S, in which the *N*-methyl­thio­formamide unit and the pyrrolidine ring mean plane are oriented at dihedral angles of 5.9 (5) and 5.9 (4)°. In the crystal, zigzag *C*(4) chains extending along the *a* axis are formed due to N—H⋯S hydrogen bonds between alternate arrangements of mol­ecules. The chains are inter­linked by C—H⋯S hydrogen bonds.

## Related literature

For a related structure, see: Jiang (2009 ▶). For graph–set notation, see: Bernstein *et al.* (1995 ▶).
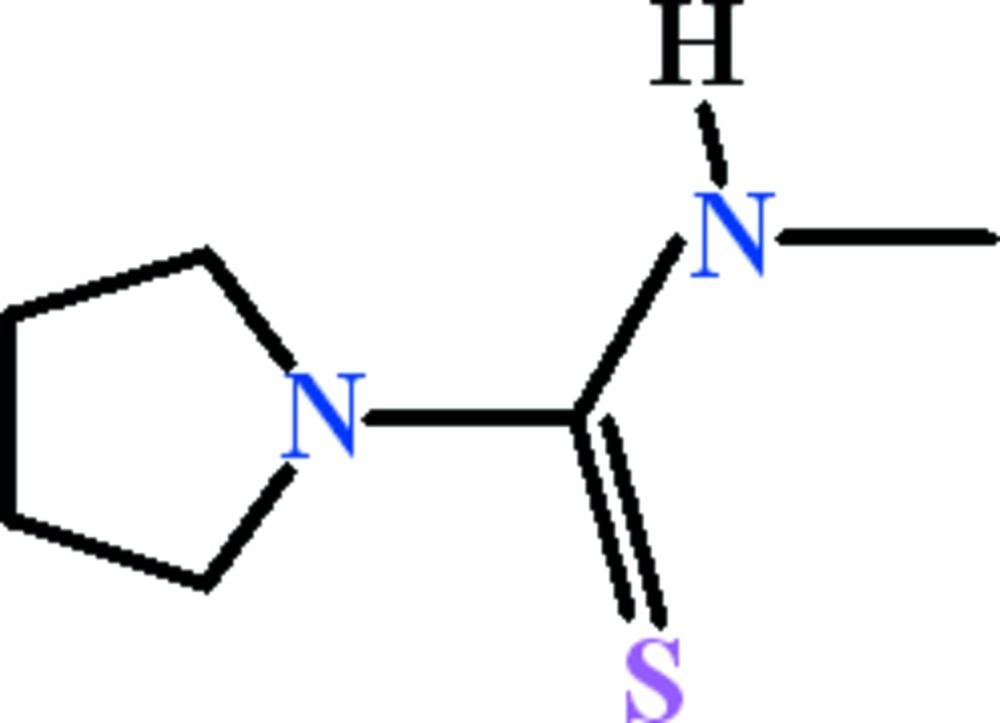


## Experimental

### 

#### Crystal data

C_6_H_12_N_2_S
*M* *_r_* = 144.25
Triclinic, 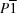

*a* = 8.616 (2) Å
*b* = 9.077 (2) Å
*c* = 10.796 (3) Å
α = 73.725 (14)°
β = 86.656 (15)°
γ = 76.177 (16)°
*V* = 787.0 (3) Å^3^
*Z* = 4
Mo *K*α radiation
μ = 0.33 mm^−1^
*T* = 296 K
0.30 × 0.25 × 0.20 mm

#### Data collection

Bruker Kappa APEXII CCD diffractometer
Absorption correction: multi-scan (*SADABS*; Bruker, 2005 ▶) *T*_min_ = 0.957, *T*_max_ = 0.966
9119 measured reflections
2699 independent reflections
1385 reflections with *I* > 2σ(*I*)
*R*_int_ = 0.067

#### Refinement

*R*[*F* ^2^ > 2σ(*F* ^2^)] = 0.079
*wR*(*F* ^2^) = 0.262
*S* = 1.04
2699 reflections
165 parameters
H-atom parameters constrained
Δρ_max_ = 0.43 e Å^−3^
Δρ_min_ = −0.33 e Å^−3^

### 

Data collection: *APEX2* (Bruker, 2007 ▶); cell refinement: *SAINT* (Bruker, 2007 ▶); data reduction: *SAINT*; program(s) used to solve structure: *SHELXS97* (Sheldrick, 2008 ▶); program(s) used to refine structure: *SHELXL97* (Sheldrick, 2008 ▶); molecular graphics: *ORTEP-3 for Windows* (Farrugia, 1997 ▶) and *PLATON* (Spek, 2009 ▶); software used to prepare material for publication: *WinGX* (Farrugia, 1999 ▶) and *PLATON*.

## Supplementary Material

Crystal structure: contains datablock(s) global, I. DOI: 10.1107/S1600536812020971/bq2355sup1.cif

Structure factors: contains datablock(s) I. DOI: 10.1107/S1600536812020971/bq2355Isup2.hkl

Supplementary material file. DOI: 10.1107/S1600536812020971/bq2355Isup3.cml

Additional supplementary materials:  crystallographic information; 3D view; checkCIF report

## Figures and Tables

**Table 1:** Hydrogen-bond geometry (Å, °)

*D*—H⋯*A*	*D*—H	H⋯*A*	*D*⋯*A*	*D*—H⋯*A*
N1—H1⋯S2^i^	0.86	2.73	3.472 (5)	145
N3—H4⋯S1^ii^	0.86	2.64	3.410 (5)	150
C12—H12*B*⋯S1^iii^	0.97	2.84	3.765 (5)	159

Symmetry codes: (i) 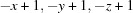; (ii) 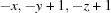; (iii) 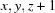.

**Fig. 1.**
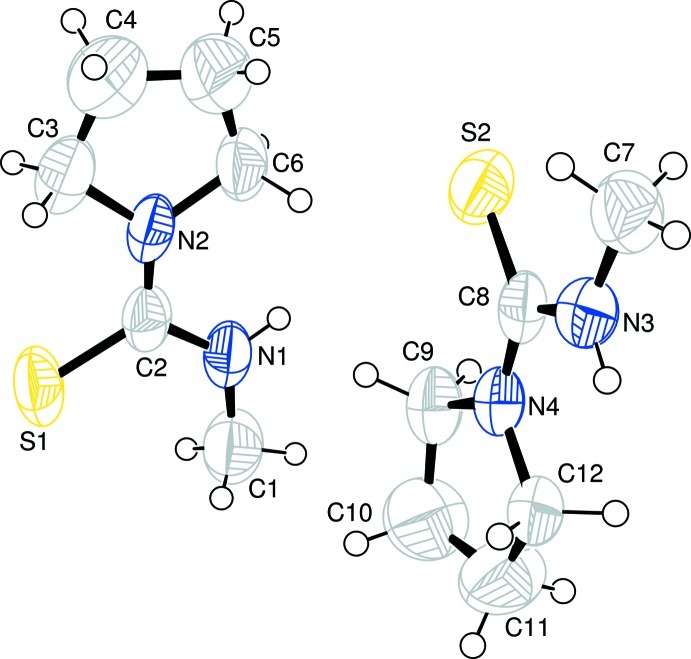
View of the title compound with the atom numbering scheme. The thermal ellipsoids are drawn at the 50% probability level.

**Fig. 2.**
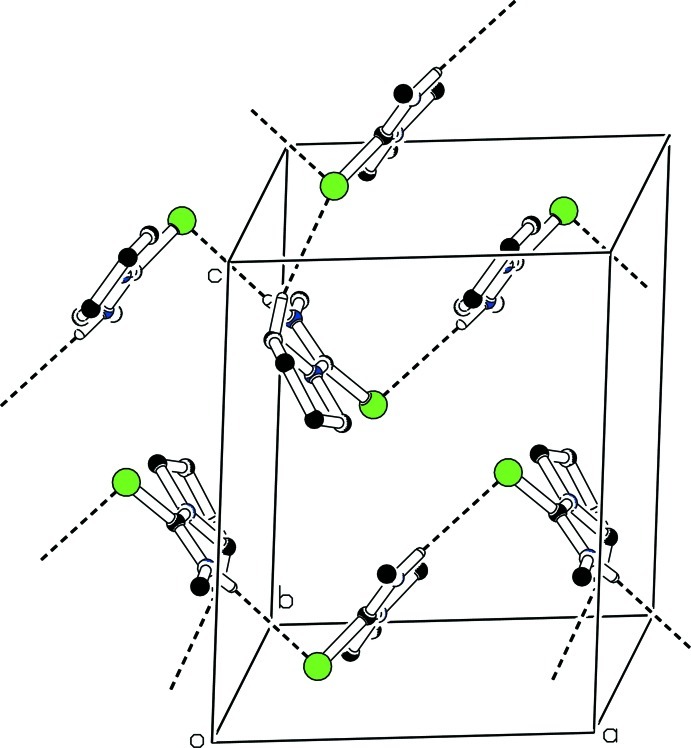
The partial packing (PLATON; Spek, 2009) which shows that molecules are interlinked to form polymeric chains along the a-axis. The H-atoms not involved in H-bonding are omitted for clarity.

## supplementary crystallographic information

### Comment

The title compound (I), (Fig. 1) has been synthesized as a derivative. The crystal structure of *N*-phenylpyrrolidine-1-carbothioamide related to this structure (I) has been published previously (Jiang, 2009). In (I), two molecules in the asymmetric unit are present, which differ slightly from each other geometrically. In one molecule, the *N*-methylthioformamide moiety A (C1/N1/C2/S1) and the pyrrolidine ring B (N2/C3–C6) are planar with r.m.s. deviation of 0.0010 Å and 0.0360 Å, respectively. The dihedral angle between A/B is 5.88 (46)°. In second molecule, the similar groups C (C7/N3/C8/S2) and D (N4/C9—C12) are also planar with r.m.s. deviation of 0.0032 Å and 0.0839 Å, respectively and the dihedral angle between C/D is 5.92 (39)°. Both molecules are interlinked through classical intramolecular H–bonding of N—H···S type (Table 1, Fig. 2) with *C*(4) chains (Bernstein *et al.*, 1995) to form zigzag infinite one-dimensional polymeric chains extending along the *a*-axis. The polymeric chains are interlinked due to C—H···S type of H–bonding (Table 1, Fig. 2).

### Experimental

A solution of pyrrolidine (0.36 g, 5.00 mmol) in CH_3_CN (3 ml) was added dropwise to a stirred solution of methyl isothiocyanate (0.47 ml, 5.50 mmol) in CH_3_CN (10 ml, anhydrous) under cooling in an ice-bath to keep the reaction temperature below 283 K. The ice-bath was removed and stirring was continued at room temperature for 2 h to furnish a yellow-colored solution. The reaction mixture was extracted with ethylacetate and subjected to column chromatography to get the colorless product in 67% yield and then recrystalized with methanol to get colorless prisms of (I).

### Refinement

The H-atoms were positioned geometrically (C–H = 0.96–0.97 Å, N—H = 0.86 Å) and refined as riding with *U*_iso_(H) = *xU*_eq_(C, N), where *x* = 1.5 for methyl and *x* = 1.2 for all other H-atoms.

### Crystal data

**Table:**

C_6_H_12_N_2_S	*Z* = 4
*M**_r_* = 144.25	*F*(000) = 312
Triclinic, *P*1	*D*_x_ = 1.217 Mg m^−^^3^
Hall symbol: -P 1	Mo *K*α radiation, λ = 0.71073 Å
*a* = 8.616 (2) Å	Cell parameters from 1204 reflections
*b* = 9.077 (2) Å	θ = 2.4–26.0°
*c* = 10.796 (3) Å	µ = 0.33 mm^−^^1^
α = 73.725 (14)°	*T* = 296 K
β = 86.656 (15)°	Prism, colorless
γ = 76.177 (16)°	0.30 × 0.25 × 0.20 mm
*V* = 787.0 (3) Å^3^	

### Data collection

**Table:**

Bruker Kappa APEXII CCD diffractometer	2699 independent reflections
Radiation source: fine-focus sealed tube	1385 reflections with *I* > 3σ(*I*)
Graphite monochromator	*R*_int_ = 0.067
Detector resolution: 8.10 pixels mm^-1^	θ_max_ = 25.0°, θ_min_ = 2.4°
ω scans	*h* = −10→10
Absorption correction: multi-scan (*SADABS*; Bruker, 2005)	*k* = −11→11
*T*_min_ = 0.957, *T*_max_ = 0.966	*l* = −13→13
9119 measured reflections	

### Refinement

**Table:**

Refinement on *F*^2^	Primary atom site location: structure-invariant direct methods
Least-squares matrix: full	Secondary atom site location: difference Fourier map
*R*[*F*^2^ > 2σ(*F*^2^)] = 0.079	Hydrogen site location: inferred from neighbouring sites
*wR*(*F*^2^) = 0.262	H-atom parameters constrained
*S* = 1.04	*w* = 1/[σ^2^(*F*_o_^2^) + (0.1268*P*)^2^ + 0.3916*P*] where *P* = (*F*_o_^2^ + 2*F*_c_^2^)/3
2699 reflections	(Δ/σ)_max_ < 0.001
165 parameters	Δρ_max_ = 0.43 e Å^−^^3^
0 restraints	Δρ_min_ = −0.33 e Å^−^^3^

### Special details

**Table:**

Geometry. All esds (except the esd in the dihedral angle between two l.s. planes) are estimated using the full covariance matrix. The cell esds are taken into account individually in the estimation of esds in distances, angles and torsion angles; correlations between esds in cell parameters are only used when they are defined by crystal symmetry. An approximate (isotropic) treatment of cell esds is used for estimating esds involving l.s. planes.
Refinement. Refinement of *F*^2^ against ALL reflections. The weighted *R*-factor *wR* and goodness of fit *S* are based on *F*^2^, conventional *R*-factors *R* are based on *F*, with *F* set to zero for negative *F*^2^. The threshold expression of *F*^2^ > σ(*F*^2^) is used only for calculating *R*-factors(gt) *etc*. and is not relevant to the choice of reflections for refinement. *R*-factors based on *F*^2^ are statistically about twice as large as those based on *F*, and *R*- factors based on ALL data will be even larger.

### Fractional atomic coordinates and isotropic or equivalent isotropic displacement parameters (Å^2^)

**Table:**

	*x*	*y*	*z*	*U*_iso_*/*U*_eq_	
C1	0.4239 (8)	0.1340 (7)	0.3083 (6)	0.101 (2)	
H1A	0.3186	0.1209	0.3350	0.151*	
H1B	0.4965	0.0862	0.3803	0.151*	
H1C	0.4586	0.0844	0.2406	0.151*	
C2	0.3360 (6)	0.3972 (7)	0.1594 (5)	0.0699 (14)	
C3	0.2574 (7)	0.6685 (8)	0.0191 (5)	0.0913 (18)	
H3A	0.2961	0.6484	−0.0622	0.110*	
H3B	0.1437	0.6732	0.0248	0.110*	
C4	0.2906 (12)	0.8143 (10)	0.0304 (9)	0.153 (3)	
H4A	0.3452	0.8611	−0.0463	0.184*	
H4B	0.1906	0.8885	0.0365	0.184*	
C5	0.3868 (11)	0.7860 (8)	0.1411 (7)	0.123 (3)	
H5A	0.3265	0.8361	0.2030	0.148*	
H5B	0.4801	0.8297	0.1166	0.148*	
C6	0.4365 (7)	0.6143 (7)	0.1987 (5)	0.0846 (17)	
H6A	0.4123	0.5869	0.2900	0.101*	
H6B	0.5503	0.5764	0.1880	0.101*	
C7	0.0612 (8)	0.8849 (7)	0.7020 (6)	0.0905 (18)	
H7A	0.1661	0.8968	0.7156	0.136*	
H7B	−0.0082	0.9109	0.7695	0.136*	
H7C	0.0199	0.9542	0.6202	0.136*	
C8	0.1664 (6)	0.6492 (6)	0.6253 (4)	0.0668 (14)	
C9	0.2534 (7)	0.4014 (7)	0.5615 (5)	0.0791 (15)	
H9A	0.3672	0.3846	0.5754	0.095*	
H9B	0.2292	0.4497	0.4704	0.095*	
C10	0.2012 (10)	0.2515 (9)	0.6076 (8)	0.126 (3)	
H10A	0.1297	0.2437	0.5447	0.151*	
H10B	0.2930	0.1627	0.6193	0.151*	
C11	0.1210 (11)	0.2487 (8)	0.7269 (7)	0.125 (3)	
H11A	0.1933	0.1864	0.7980	0.150*	
H11B	0.0307	0.2009	0.7314	0.150*	
C12	0.0651 (7)	0.4120 (6)	0.7365 (5)	0.0768 (15)	
H12A	−0.0481	0.4515	0.7165	0.092*	
H12B	0.0846	0.4183	0.8222	0.092*	
N1	0.4210 (5)	0.2971 (5)	0.2626 (4)	0.0771 (13)	
H1	0.4775	0.3348	0.3037	0.093*	
N2	0.3437 (5)	0.5463 (6)	0.1280 (4)	0.0739 (12)	
N3	0.0697 (5)	0.7250 (5)	0.7032 (4)	0.0746 (12)	
H4	0.0100	0.6740	0.7564	0.089*	
N4	0.1603 (5)	0.5002 (5)	0.6404 (4)	0.0677 (11)	
S1	0.22218 (19)	0.3304 (2)	0.07310 (14)	0.0932 (6)	
S2	0.28575 (19)	0.74016 (18)	0.51593 (13)	0.0881 (6)	

### Atomic displacement parameters (Å^2^)

**Table:**

	*U*^11^	*U*^22^	*U*^33^	*U*^12^	*U*^13^	*U*^23^
C1	0.122 (5)	0.099 (5)	0.083 (4)	−0.026 (4)	−0.013 (4)	−0.024 (4)
C2	0.071 (3)	0.102 (4)	0.040 (3)	−0.018 (3)	0.003 (2)	−0.027 (3)
C3	0.087 (4)	0.116 (5)	0.060 (3)	−0.020 (4)	−0.009 (3)	−0.007 (3)
C4	0.202 (10)	0.116 (6)	0.127 (7)	−0.046 (6)	−0.058 (7)	0.007 (5)
C5	0.180 (8)	0.100 (5)	0.095 (5)	−0.040 (5)	−0.017 (5)	−0.025 (4)
C6	0.102 (4)	0.107 (5)	0.052 (3)	−0.030 (4)	−0.004 (3)	−0.027 (3)
C7	0.115 (5)	0.085 (4)	0.078 (4)	−0.024 (3)	0.000 (3)	−0.032 (3)
C8	0.071 (3)	0.088 (4)	0.042 (3)	−0.018 (3)	−0.014 (2)	−0.016 (3)
C9	0.081 (4)	0.100 (4)	0.061 (3)	−0.013 (3)	−0.001 (3)	−0.035 (3)
C10	0.154 (7)	0.104 (5)	0.136 (7)	−0.036 (5)	0.023 (6)	−0.061 (5)
C11	0.187 (8)	0.088 (5)	0.109 (6)	−0.044 (5)	0.038 (5)	−0.036 (4)
C12	0.093 (4)	0.096 (4)	0.050 (3)	−0.038 (3)	0.000 (3)	−0.021 (3)
N1	0.088 (3)	0.093 (3)	0.054 (3)	−0.023 (3)	−0.011 (2)	−0.022 (2)
N2	0.081 (3)	0.098 (3)	0.041 (2)	−0.020 (3)	−0.009 (2)	−0.017 (2)
N3	0.090 (3)	0.086 (3)	0.055 (3)	−0.031 (2)	0.008 (2)	−0.023 (2)
N4	0.083 (3)	0.080 (3)	0.045 (2)	−0.021 (2)	−0.001 (2)	−0.023 (2)
S1	0.0962 (12)	0.1408 (15)	0.0594 (9)	−0.0374 (10)	−0.0056 (8)	−0.0447 (9)
S2	0.0935 (12)	0.1108 (13)	0.0601 (9)	−0.0357 (9)	0.0018 (8)	−0.0135 (8)

### Geometric parameters (Å, º)

**Table:**

C1—N1	1.418 (7)	C7—H7A	0.9600
C1—H1A	0.9600	C7—H7B	0.9600
C1—H1B	0.9600	C7—H7C	0.9600
C1—H1C	0.9600	C8—N4	1.330 (6)
C2—N2	1.316 (6)	C8—N3	1.358 (6)
C2—N1	1.346 (6)	C8—S2	1.688 (5)
C2—S1	1.705 (5)	C9—N4	1.475 (6)
C3—C4	1.457 (9)	C9—C10	1.480 (9)
C3—N2	1.464 (7)	C9—H9A	0.9700
C3—H3A	0.9700	C9—H9B	0.9700
C3—H3B	0.9700	C10—C11	1.422 (10)
C4—C5	1.423 (10)	C10—H10A	0.9700
C4—H4A	0.9700	C10—H10B	0.9700
C4—H4B	0.9700	C11—C12	1.476 (8)
C5—C6	1.473 (8)	C11—H11A	0.9700
C5—H5A	0.9700	C11—H11B	0.9700
C5—H5B	0.9700	C12—N4	1.462 (6)
C6—N2	1.474 (6)	C12—H12A	0.9700
C6—H6A	0.9700	C12—H12B	0.9700
C6—H6B	0.9700	N1—H1	0.8600
C7—N3	1.432 (6)	N3—H4	0.8600
			
N1—C1—H1A	109.5	N4—C8—N3	115.8 (5)
N1—C1—H1B	109.5	N4—C8—S2	122.6 (4)
H1A—C1—H1B	109.5	N3—C8—S2	121.6 (4)
N1—C1—H1C	109.5	N4—C9—C10	103.7 (5)
H1A—C1—H1C	109.5	N4—C9—H9A	111.0
H1B—C1—H1C	109.5	C10—C9—H9A	111.0
N2—C2—N1	118.2 (4)	N4—C9—H9B	111.0
N2—C2—S1	121.6 (4)	C10—C9—H9B	111.0
N1—C2—S1	120.2 (4)	H9A—C9—H9B	109.0
C4—C3—N2	104.4 (5)	C11—C10—C9	108.4 (6)
C4—C3—H3A	110.9	C11—C10—H10A	110.0
N2—C3—H3A	110.9	C9—C10—H10A	110.0
C4—C3—H3B	110.9	C11—C10—H10B	110.0
N2—C3—H3B	110.9	C9—C10—H10B	110.0
H3A—C3—H3B	108.9	H10A—C10—H10B	108.4
C5—C4—C3	111.2 (6)	C10—C11—C12	108.9 (6)
C5—C4—H4A	109.4	C10—C11—H11A	109.9
C3—C4—H4A	109.4	C12—C11—H11A	109.9
C5—C4—H4B	109.4	C10—C11—H11B	109.9
C3—C4—H4B	109.4	C12—C11—H11B	109.9
H4A—C4—H4B	108.0	H11A—C11—H11B	108.3
C4—C5—C6	108.3 (6)	N4—C12—C11	103.6 (5)
C4—C5—H5A	110.0	N4—C12—H12A	111.0
C6—C5—H5A	110.0	C11—C12—H12A	111.0
C4—C5—H5B	110.0	N4—C12—H12B	111.0
C6—C5—H5B	110.0	C11—C12—H12B	111.0
H5A—C5—H5B	108.4	H12A—C12—H12B	109.0
C5—C6—N2	104.9 (5)	C2—N1—C1	124.6 (5)
C5—C6—H6A	110.8	C2—N1—H1	117.7
N2—C6—H6A	110.8	C1—N1—H1	117.7
C5—C6—H6B	110.8	C2—N2—C3	124.3 (5)
N2—C6—H6B	110.8	C2—N2—C6	125.1 (4)
H6A—C6—H6B	108.8	C3—N2—C6	110.6 (5)
N3—C7—H7A	109.5	C8—N3—C7	123.9 (5)
N3—C7—H7B	109.5	C8—N3—H4	118.0
H7A—C7—H7B	109.5	C7—N3—H4	118.0
N3—C7—H7C	109.5	C8—N4—C12	125.5 (4)
H7A—C7—H7C	109.5	C8—N4—C9	123.2 (4)
H7B—C7—H7C	109.5	C12—N4—C9	111.3 (4)
			
N2—C3—C4—C5	−1.7 (10)	C4—C3—N2—C6	−4.0 (7)
C3—C4—C5—C6	6.6 (11)	C5—C6—N2—C2	−170.9 (6)
C4—C5—C6—N2	−8.6 (8)	C5—C6—N2—C3	7.8 (6)
N4—C9—C10—C11	−15.4 (8)	N4—C8—N3—C7	179.3 (5)
C9—C10—C11—C12	21.4 (10)	S2—C8—N3—C7	−1.0 (7)
C10—C11—C12—N4	−17.9 (8)	N3—C8—N4—C12	−3.0 (7)
N2—C2—N1—C1	179.6 (5)	S2—C8—N4—C12	177.3 (4)
S1—C2—N1—C1	−0.3 (7)	N3—C8—N4—C9	178.1 (4)
N1—C2—N2—C3	−179.7 (5)	S2—C8—N4—C9	−1.6 (6)
S1—C2—N2—C3	0.3 (7)	C11—C12—N4—C8	−170.9 (5)
N1—C2—N2—C6	−1.1 (8)	C11—C12—N4—C9	8.1 (6)
S1—C2—N2—C6	178.8 (4)	C10—C9—N4—C8	−176.9 (5)
C4—C3—N2—C2	174.7 (6)	C10—C9—N4—C12	4.1 (6)

### Hydrogen-bond geometry (Å, º)

**Table:**

*D*—H···*A*	*D*—H	H···*A*	*D*···*A*	*D*—H···*A*
N1—H1···S2^i^	0.86	2.73	3.472 (5)	145
N3—H4···S1^ii^	0.86	2.64	3.410 (5)	150
C12—H12*B*···S1^iii^	0.97	2.84	3.765 (5)	159

Symmetry codes: (i) −*x*+1, −*y*+1, −*z*+1; (ii) −*x*, −*y*+1, −*z*+1; (iii) *x*, *y*, *z*+1.
